# The Expression of SOCS and NF-ϰB p65 in Hypopharyngeal Carcinoma

**Published:** 2018-12

**Authors:** Shu FENG, Junfu WANG, Xiaoqun XU, Xuemei CHEN, Junwen LUAN, Qinghong SU, Meng LUAN, Huali WANG, Changsheng ZHAO

**Affiliations:** 1. Dept. of Clinical Laboratory, Shandong Provincial Hospital, Jinan, Shandong, China; 2. Institute of Basic Medicine, Shandong Academy of Medical Sciences, Jinan, Shandong, China; 3. Dept. of Otolaryngology, The Second Hospital of Shandong University, Jinan, Shandong, China; 4. Dept. of Gynaecology, The Second Hospital of Shandong University, Jinan, Shandong, China; 5. Dept. of Nutriology, The Second Hospital of Shandong University, Jinan, Shandong, China

**Keywords:** Hypopharyngeal carcinoma, Suppressor of cytokine signaling (SOCS)-1, SOCS-3, Nuclear factor (NF)-ϰB

## Abstract

**Background:**

Hypopharyngeal carcinoma is one of the most common types of head and neck tumors. Suppressers of cytokine signalling (SOCS) family members are key regulators of cytokine homeostasis, they play important roles in the process of cell proliferation, differentiation, maturation and apoptosis, and participate in the occurrence and development of tumor. The abnormal activation of NF-ϰB is an important feature of the tumor. The aim of this study was to investigate the relationships among SOCS, NF-ϰB p65 and hypopharyngeal carcinoma development.C

**Methods:**

We included 72 hypopharyngeal cancer patients and 9 swallow cyst patients. The patients were recruited at The Second Hospital of Shandong University (Jinan, China) between 2014 and 2016. The mRNA and protein expression levels of SOCS-1, SOCS-3 and NF-ϰB p65 in hypopharyngeal carcinoma tissues, para-cancerous tissues and control tissues were detected by RT-PCR and Western blot analysis, respectively.

**Results:**

Hypopharyngeal carcinoma tissues had lower level expression of SOCS-1 and SOCS-3 than pericarcinoma tissues, but there was no significant difference, while cancer tissues had significantly higher level expression of NF-ϰB p65 than that of pericarcinoma tissues (0.412±0.266, 0.281±0.231, t=2.969, *P*=0.004). The early stage patients had striking higher level expression of SOCS-1 and SOCS-3 than that in advanced stages (F=16.202, *P*<0.001; F=52.295, *P*<0.001), while the expression of NF-ϰB p65 in early stages had lower level than that in advanced stages (F=3.383, *P*=0.04).

**Conclusion:**

SOCS-1, SOCS-3 may be protective factors while NF-ϰB p65 could be a harmful factor in hypopharyngeal carcinoma.

## Introduction

Head and neck tumor is one of the main cancers endangering human health, ranking the sixth most common cancer in the world (1). Hypopharyngeal carcinoma is one of the most common types of head and neck tumors, which remains a highly lethal disease and serious threat to human life. However, there are a variety of treatments, but the prognosis is not ideal.

Currently, some of the inflammation and the incidence of tumor have a close relationship, researches of immunity, inflammation, and cancer are being gradually concerned. Chronic inflammation can promote the occurrence and development of cancer, participate in various pathological processes and is involved in the growth and migration of tumors. Pro-inflammatory cytokines and its downstream signaling pathway play key roles in inflammation and tumors. “Suppressers of cytokine signalling (SOCS) constitute a newly identified family of proteins which negatively regulate cytokine signal transduction”(2). The SOCS family consists of eight members (SOCS1-7 and CIS) (3), each has a central SH2 domain, an amino-terminal domain of variable length and sequence, and a conserved SOCS box. SOCS proteins can be induced by several cytokines in various tissues in a tissue-specific manner (4). Once induced, they can inhibit cytokine-mediated Janus kinase/Signal transducer and activator of transcription (JAK/STAT) activation, thereby forming a negative regulatory feedback loop. SOCS-1 and SOCS-3 are the main inhibitors of JAK/STAT3 signaling pathway and they have similar structural characteristics (5, 6). SOCS proteins play important roles in a variety of tumors, but it was not yet clear in hypopharyngeal carcinoma.

Nuclear factor (NF)-ϰB was first discovered by Sen and Baltimore in 1986. As an important transcription factor, NF-ϰB played an irreplaceable role in the life activities of cells(7). “The mammalian NF-ϰB /Rel family contains 5 members: RelA (p65), NF-ϰB 1 (p50/p105), NF-ϰB 2 (p52/p100), c-Rel and RelB”(8). NF-ϰB was activated via two signaling pathways, which were the classical pathway and non-classical pathway, played a pivotal role in inflammation and tumors. In the present study, the mRNA and protein expressions of SOCS-1, SOCS-3 and NF-ϰB p65 were analyzed at the tissue level by reverse transcription-polymerase chain reactions (RT-PCR) and western blot analysis to investigate the function and clinical significance of SOCS-1, SOCS-3 and NF-ϰB p65 in hypopharyngeal carcinoma and their involvement in hypopharyngeal carcinoma pathogenesis.

## Materials and Methods

### Patients and samples

The present study contains 72 patients with a mean age of 60.42 ± 8.59 yr (range 47–77 yr) histologically diagnosed with hypopharyngeal carcinoma and 9 swallow cyst patients, with a mean age of 65.78±10.24 yr (range, 50–79 yr), that served as age- and gender-matched controls. The patients were recruited at The Second Hospital of Shandong University (Jinan, China) between 2014 and 2016.

The staging of the tumor was determined in accordance with the American Joint Committee on Cancer (AJCC), tumor-node-metastasis (TNM) classification. None of the patients had received any chemotherapy, radiation therapy or immuno-therapy within 2 months prior to surgery. Patients with any other chronic diseases such as tuberculosis, diabetes, autoimmune diseases or other malignant tumors were excluded.

The protocol was approved by the Ethics committee of Shandong University School of Medicine, and all of the patients involved in this study signed informed consent. Cancer tissues, precancerous tissues and normal control tissues were identified by stereoscopy and quickly frozen sectioning. Two tissue sections were collected and snap-frozen for RNA extraction and protein preparation.

### Main reagent

The total RNA extraction kit was purchased from Transgen Biotech Company (Beijing, China). MMLV reverse transcriptase and Taq DNA polymerase were purchased from Promega Corporation (Madison, WI, USA). PCR primers for the detection of SOCS-1, SOCS-3, NF-κB p65 and β-actin mRNA were designed using the OLIGO Primer Analysis Software, version 5.0 (NBA, Software and Research Services for Tomorrow’s Discoveries, National Biosciences, Plymouth, MN, USA) (Table 1).

**Table 1:** SOCS-1, SOCS-3, NF-κB p65 and β-actin primer sequence for RT-PCR

***Aim gene***	***Oligonucleotide sequence***	***Product size(bp)***
SOCS-1	(F) 5′TTGGAGGGAGCGGATGGGTGTAG 3′(R)5′AGAGGTAGGAGGTGCGAGTTCAGGTC 3′	185
SOCS-3	(F) 5′GTCACCCACAGCAAGTTTCC 3′(R) 5′CCGACAGAGATGCTGAAGAG 3′	579
NF-κB (p65)	(F) 5′TGCTGTGCGGCTCTGCTTCC 3′(R) 5′AGGCTGGGGTCTGCGTAGGG 3′	321
β-actin	(F) 5′GTGGGCGCCCAGGCACCA3′(R) 5′CTCCTTAATGTCACGCACGATTT3′	539

### RT-PCR

RNA was extracted from tissues using the guanidine thiocyanate phenol-chloroform method. The quality of the RNA yield was assessed by electrophoresis on a 1.5% agarose gel in 0.5 mol Tris/Borate/EDTA buffer. The optical density of the RNA samples was measured and samples exhibiting an A260–A280 ratio of 1.8–2.0 were used to obtain cDNA. RT-PCR was performed using a RNA PCR kit (Perkin-Elmer, Norwalk, CT, USA). The relative intensity (RI) of each band was determined according to the following equation: RI=density of target gene/density of β-actin. To exclude the possibility of contamination, reactions containing RT-PCR reagents including cytokine PCR primers without sample RNA were used as the negative control groups.

### Western blot analysis

SDS-PAGE and immunoblotting were performed according to standard techniques. Briefly, the prepared tissues (100mg) were lysed at 4 °C for 30 min in lysis buffer (Beijing Leagene Biotech. Co, Ltd, Beijing, China). The lysates were centrifuged at 15000 rpm for 20 min at 4 °C to remove nuclei and undisrupted tissues. Protein concentration was determined using Bio-Rad protein assay solution (Bio-Rad Laboratories, Inc., Hercules, CA, USA) with bovine serum albumin as the standard. The protein samples were boiled for 10 min and loaded onto a 14% SDS-PAGE gel followed by electrophoresis for 2 h. The proteins were electrophoretically transferred onto a 0.22 μm nitrocellulose membrane. The nitrocellulose membrane was blocked with 5% skim milk at room temperature for 1 h, and immunoblotted with monoclonal mouse anti-human SOCS-1, SOCS-3, rabbit anti-human NF-κB p65 and polyclonal rabbit β-actin primary antibodies. After the membrane was washed three times at 5-min intervals in PBS-T, the membrane was subsequently incubated with goat anti-mouse IgG-HRP or goat anti-rabbit IgG-HRP diluted to 1:5000 for 1 h at room temperature. After the membrane was washed three times at 5-min intervals PBS-T, and the immunoblots were then visualized using a LAS4000 Chemiluminescence Imager (Fijifilm, Tokyo, Japan) with associated software. For presentation, immunoblots were opened in PhotoShop CS2 (Adobe Systems, Mountain View, CA, USA).

### Statistical analysis

To determine the levels of SOCS-1, SOCS-3 and NF-κB p65 in hypopharyngeal carcinoma, data analysis was performed using SPSS 17.0 statistical software (Chicago, IL, USA). Data were presented as the mean ± standard deviation. The paired samples t-test was used to compare differences between hypopharyngeal carcinoma and pericarcinoma tissues. One-way analysis of variance analysis was used to compare the differences between groups at different clinical stages. *P*<0.05 was considered to indicate a statistically significant difference.

## Results

### Patient clinicopathological characteristics

The clinicopathological characteristics of the patient cohort, which included 72 hypopharyngeal carcinoma patients and 9 swallow cyst controls are shown in Table 2. Of the 72 hypopharyngeal carcinoma patients, 69 (95.83%) were male and only 3 (4.17%) were female, male to female ratio up to 23. In accordance with TNM classification, the majority of patients presented with large tumors (T3 + T4; 66.7%) and lymph node involvement (N+; 75.00%). The majority of patients exhibited advanced stage disease (stage III + IV; 91.67%), while 8.33% exhibited early stage cancer (stage I + II). All the patients were pathologically diagnosed with squamous cell carcinoma. Histologically, 62.50% of patients exhibited poorly- or moderately-differentiated tumors.

**Table 2:** Clinic pathological characteristics of study subjects

***Characteristics***	***No. (%)***	
	***Patients***	***Control***
Age (yr)		
Range	47–77	50–79
Mean±SD	60.42±8.59	65.78±10.24
Gender		
Male	69(95.83)	
Female	3 (4.17)	8
Tumor Size		1
T1+T2	24 (33.33)	
T3	42 (58.34)	
T4	6 (8.33)	
Lymph node involvement		
N0	18(25.00)	
N+	54(75.00)	
Pathological classification		
Squamous cell carcinoma	72 (100.0)	
Histological classification Well differentiated	9 (12.50)	
Moderately differentiated	33 (45.83)	
Poorly differentiated	30 (41.67)	
Clinical stage		
I+II	6(8.33)	
III	36(50.00)	
IV	30 (41.67)	

### The lower expression of SOCS in hypopharyngeal carcinoma

**Fig. 1:**
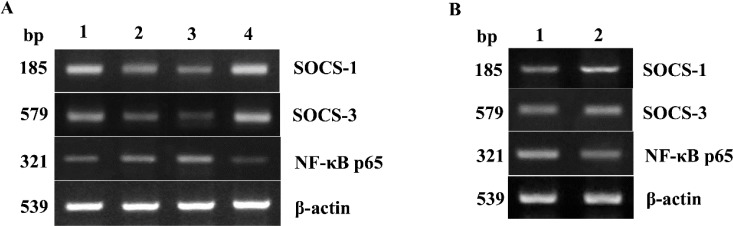
mRNA expression of SOCS-1, SOCS-3 and NF-ϰB p65 in tumor tissues of different clinical stages and pericarcinoma tissues (A) Representative mRNA expression of SOCS-1, SOCS-3 and NF-ϰB p65 in tumor tissues of different clinical stages and normal control tissues. (Lane 1, stage I +II patient; lane 2, stage III patient; lane 3, stage IV patient; lane 4, normal control). (B) Representative mRNA expression of SOCS-1, SOCS-3 and NF-ϰB p65 in tumor tissues and pericarcinoma tissues. (Lane 1, tumor tissue; lane 2, pericarcinoma tissue)

**Fig. 2:**
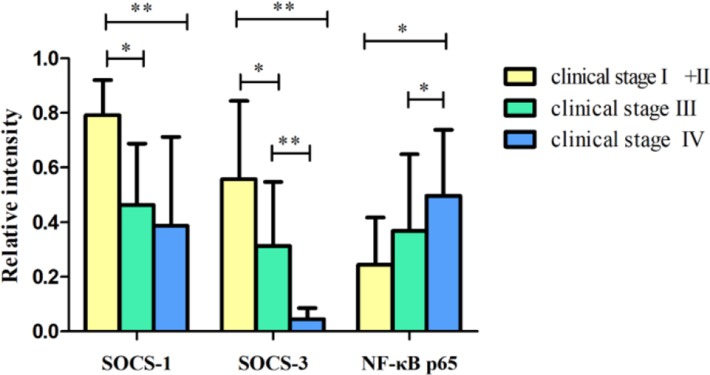
Relative intensity of SOCS-1, SOCS-3 and NF-ϰB p65 mRNA expression in tumor tissues of different clinical stages and normal control tissues Early stage patients exhibited higher levels of SOCS-1 and SOCS-3 mRNA expression than advanced stage patients. The expression of NF-ϰB p65 was lower in early stage patients compared with advanced stage patients. (^*^P<0.05 and ^**^P<0.01)

**Fig. 3:**
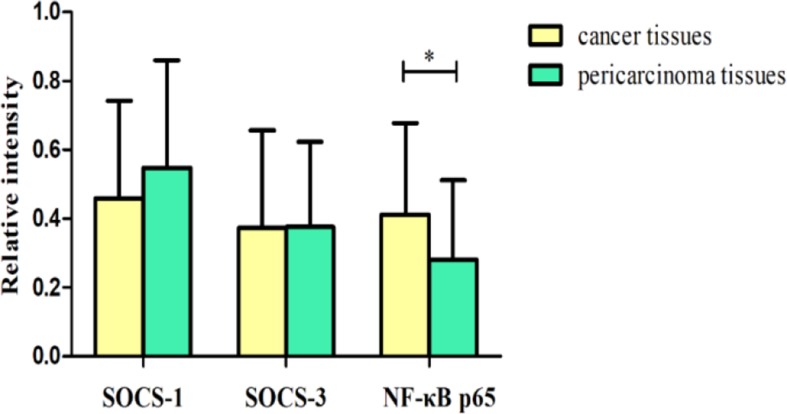
Relative intensity of SOCS-1, SOCS-3 and NF-ϰB p65 mRNA expression in hypopharyngeal carcinoma tissues and pericarcinoma tissues The intensity of SOCS-1 and SOCS-3 expression was lower in hypopharyngeal carcinoma tissues than pericarcinoma tissues. The intensity of NF-ϰB p65 expression was higher in hypopharyngeal carcinoma tissues than in pericarcinoma tissues. (*P<0.05)

Total RNA of 72 fresh tissues obtained from hypopharyngeal carcinoma patients and 9 mucosal tissues obtained from swallow cyst control patients were prepared. The cytokine mRNA expression profiles from the aforementioned tissues were analyzed by RT-PCR. The mRNA expressions of SOCS-1 and SOCS-3 in tissues were analyzed. The SOCS expression was higher in the control tissues than that in hypopharyngeal carcinoma tissues (Figs. 1–3). Of the 72 patients, 63 cancer tissues (87.5%) and 54 pericarcinoma tissues (75.0%) expressed SOCS-1 mRNA (RI, 0.459±0.284 and 0.548±0.312, respectively; t=1.957, *P*=0.054). Overall, 66 cancer tissues (91.7%) and 48 pericarcinoma tissues (66.7%) expressed SOCS-3 mRNA (RI, 0.373±0.284 and 0.376±0.247, respectively; t=0.067, *P*=0.947). Cancer tissues exhibited a lower level mRNA expression of SOCS-1 and SOCS-3 than percarcinoma tissues, however this difference was not statistically significant. SOCS-1 and SOCS-3 mRNA expressions were significantly higher in early-stage patients than those in advanced stage patients (F=16.202, *P*<0.001 and F=52.295, *P*<0.001), especially in stage I+II was obviously higher than that in IV. The expression of SOCS-1 and SOCS-3 was decreased with clinical stages.

### NF-ϰB p65 presents dominant expression in hypopharyngeal carcinoma

The NF-ϰB p65 mRNA expression in tissues was analyzed. The expression capacity of hypopharyngeal carcinoma patients for NF-ϰB p65 was at a relatively high level. However, in the control tissues, the mRNA expression of NF-ϰB p65 was weaker (Figs. 1–3). Of the 72 patients, 63 cancer tissues (87.5%) and 57 pericarcinoma tissues (79.2%) expressed NF-ϰB p65 mRNA (RI, 0.412±0.266; and 0.281±0.231, respectively; t=2.969, *P*=0.004). The NF-ϰB p65 mRNA expression was tended to elevate with increasing clinical stages. The advanced stages patients had significantly higher level mRNA expression of NF-ϰB p65 than that in early stages (F=3.383, *P*=0.04) . Especially in clinical stage IV was significantly higher than that in clinical stage I+II (RI, 0.497±0.242 and 0.245±0.173, respectively; *P*=0.032). Cancer tissues had significantly higher level mRNA expression of NF-ϰB p65 than percarcinoma tissues (*P*=0.004).

### Immunoblotting revealed the protein expression levels of associated cytokines in hypopharyngeal carcinoma

**Fig. 4:**
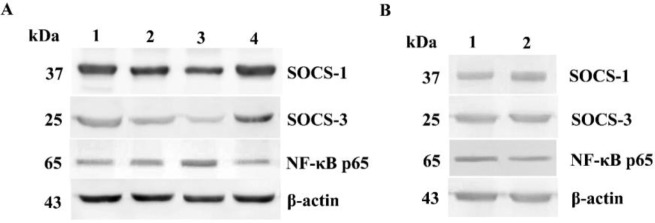
Protein expression of SOCS-1, SOCS-3 and NF-ϰB p65 in tumor tissues of different clinical stages and pericarcinoma tissues (A) Representative protein expression of SOCS-1, SOCS-3 and NF-ϰB p65 in tumor tissues of different clinical stages and normal control tissues. (Lane 1, stage I + II patient; lane 2, stage III patient; lane 3, stage IV patient; lane 4, normal control). (B) Representative protein expression of SOCS-1, SOCS-3 and NF-ϰB p65 in tumor tissues and pericarcinoma tissues. (Lane 1, tumor tissue; lane 2, pericarcinoma tissue)

The protein expressions of SOCS and NF-ϰB p65 were analyzed by western blot analysis. The protein expression level of SOCS and NF-ϰB p65 was consistent with the mRNA expression in hypopharyngeal carcinoma and pericarcinoma tissues. The protein expression of SOCS in cancer tissues was weaker than that in control tissues, but the NF-ϰB p65 protein expression was stronger in cancer tissues. SOCS-1 and SOCS-3 protein expressions in advanced stages (III + IV) were significantly lower than those in early stages (I+II). The cancer tissues expressions were lower than the pericarcinoma tissues. NF-ϰB p65 protein expression was very strong in hypopharyngeal carcinoma tissues. Its expression in advanced stage was higher than that in early stage. The cancer tissues expression was higher than the pericarcinoma tissues (Fig. 4).

## Discussion

SOCS-1 and SOCS-3 mRNA levels and protein levels were lower in hypopharyngeal carcinoma tissues than in pericarcinoma tissues, and the advanced stage patients had significantly lower level mRNA expression of SOCS-1 and SOCS-3 than those at early stages. However, NF-κB p65 expression level in early stages was significantly lower than that in advanced stages.

There are many signaling pathways regulating cell growth, proliferation, differentiation and apoptosis, if these signals have abnormalities, they will lead to carcinogenesis. Thus, the important factors in these signaling pathways are becoming target keys in anticancer drug screening and cancer therapy. Currently, a number of molecules, which can prevent oncogenic signals, have been successfully used in clinical cancer treatment. Using of cell signaling pathways will find ways to interdict the development of cancer, and bring new opportunities to the diagnosis, monitoring and treatment of cancer.

The SOCS proteins act as classic negative feedback inhibitors, which induced by cytokines, and then regulating the function of these cytokines. SOCS-1 plays a key role in the negative regulation of TLR-mediated signaling, involved in innate immunity and adaptive immunity (9, 10). SOCS proteins may inhibit the intracellular signal transduction pathways through a variety of mechanisms, such as binding to JAKs and directly inhibiting their kinase activity, blocking the combination of STATs and receptor-binding sites and inducing ubiquitination of signalling proteins (11). The N-terminal domains of SOCS-1 and SOCS-3 are also known as kinase inhibitory regions (KIR), which are responsible for inhibiting JAKs. Furthermore, the SH2 domain of them may be involved in the direct inhibition of JAKs activity (12–14). SOCS-1, SOCS-3 inhibiting JAKs and regulating tumor-associated inflammation, then inhibit a variety of tumor development and invasion. SOCS protein is STAT pathway inhibitor and can reduce cell sensitivity of cytokines (15). In many tumors, SOCS proteins inhibited the growth of tumor cells and induced apoptosis by inhibiting the STAT pathway, especially SOCS-1 and SOCS-3 (16, 17). Many accumulating pieces of evidence indicated that SOCS had low expression in various tumors, including lung cancer, gastric cancer, hepatocellular carcinoma, prostate cancer, multiple myeloma, pancreatic cancer, leukemia and lymphoma (18–20). SOCS-1, SOCS-3 may have protective functions in tumor development. Recently, new data shows different roles of these two cytokines in tumors. SOCS-1 had higher expression in breast cancer and melanoma. SOCS-1 and SOCS-3 may promote cancer development through promoting the proliferation of cancer cell.

In the present study, the mRNA expressions of SOCS-1 and SOCS-3 in hypopharyngeal carcinoma tissues were lower than that in pericarcinoma tissues, but there was no significant difference. However, SOCS-1 and SOCS-3 mRNA expression were significantly higher in early-stage patients than those in advanced stage patients, especially in stage I+II was obviously higher than that in IV. Tumor size, lymph node metastasis, and pathological classification were no associations with expression levels of SOCS-1 and SOCS-3 in our research. SOCS-1 and SOCS-3 may function as a tumor suppressor in hypopharyngeal carcinoma.

NF-ϰB family is an inducible transcription factor protein that can modulate a variety of immune functions. Normally, NF-ϰB is inactivated by the IϰB-α inhibition. When IϰB-α was phosphorylated by the upstream kinase, it would induce IϰB-α subsequent ubiquitination, and then lead to degradation of IϰB-α and activation of the NF-ϰB signaling. Persistent activation of NF-ϰB facilitate tumor development through a variety of mechanisms, such as regulation of tumor cell proliferation, inhibition apoptosis of tumor cells, promotion cells adhesion and promotion tumor angiogenesis (21). NF-ϰB was constitutively activated in multiple tumors such as ovarian cancer, lymphoma, and leukemia (22).

In our research, the advanced stage patients had significantly higher level mRNA expression of NF-ϰB than that in early stages. Hypopharyngeal carcinoma tissues have higher mRNA levels than pericarcinoma tissues. NF-ϰB activation may be a common characteristic of malignant tumor.

## Conclusion

MRNA and protein levels of the SOCS-1 and SOCS-3 were reduced associated with increased of clinical stages, however, the NF-ϰB p65 expression was tended to elevate with clinical stages. The results of SOCS-1, SOCS-3, and NF-ϰB are available on the analysis of the pathogenesis of hypopharyngeal carcinoma. This may provide a new direction for the targeted therapy of hypopharyngeal carcinoma.

## Ethical considerations

Ethical issues (Including plagiarism, informed consent, misconduct, data fabrication and/or falsification, double publication and/or submission, redundancy, etc.) have been completely observed by the authors.
